# Effects of methylation-sensitive enzymes on the enrichment of genic SNPs and the degree of genome complexity reduction in a two-enzyme genotyping-by-sequencing (GBS) approach: a case study in oil palm (*Elaeis guineensis*)

**DOI:** 10.1007/s11032-016-0572-x

**Published:** 2016-11-10

**Authors:** Wirulda Pootakham, Chutima Sonthirod, Chaiwat Naktang, Nukoon Jomchai, Duangjai Sangsrakru, Sithichoke Tangphatsornruang

**Affiliations:** National Center for Genetic Engineering and Biotechnology, 113 Thailand Science Park, Pathum Thani, 12120 Thailand

**Keywords:** SNP discovery, SNP genotyping, Genotyping-by-sequencing (GBS), Methylation-sensitive enzyme, Reduced representation library, Genome complexity reduction, Genic SNP enrichment, Oil palm (*Elaeis guineensis*)

## Abstract

Advances in next generation sequencing have facilitated a large-scale single nucleotide polymorphism (SNP) discovery in many crop species. Genotyping-by-sequencing (GBS) approach couples next generation sequencing with genome complexity reduction techniques to simultaneously identify and genotype SNPs. Choice of enzymes used in GBS library preparation depends on several factors including the number of markers required, the desired level of multiplexing, and whether the enrichment of genic SNP is preferred. We evaluated various combinations of methylation-sensitive (*Aat*II, *Pst*I, *Msp*I) and methylation-insensitive (*Sph*I, *Mse*I) enzymes for their effectiveness in genome complexity reduction and enrichment of genic SNPs. We discovered that the use of two methylation-sensitive enzymes effectively reduced genome complexity and did not require a size selection step. On the contrary, the genome coverage of libraries constructed with methylation-insensitive enzymes was quite high, and the additional size selection step may be required to increase the overall read depth. We also demonstrated the effectiveness of methylation-sensitive enzymes in enriching for SNPs located in genic regions. When two methylation-insensitive enzymes were used, only 16% of SNPs identified were located in genes and 18% in the vicinity (± 5 kb) of the genic regions, while most SNPs resided in the intergenic regions. In contrast, a remarkable degree of enrichment was observed when two methylation-sensitive enzymes were employed. Almost two thirds of the SNPs were located either inside (32–36%) or in the vicinity (28–31%) of the genic regions. These results provide useful information to help researchers choose appropriate GBS enzymes in oil palm and other crop species.

**Electronic supplementary material:**

The online version of this article (doi:10.1007/s11032-016-0572-x) contains supplementary material, which is available to authorized users.

In recent years, single nucleotide polymorphisms (SNPs) have gained a lot of interest in the scientific and breeding communities. The ubiquity of SNPs in eukaryotic genomes and their usefulness as genetic markers have been well established over the last decade (Rafalski 2002). The development of next generation sequencing technologies has facilitated rapid and inexpensive analysis of the genome sequences that have not been extensively characterized. Several research groups have successfully reported the application of next generation sequencing in SNP discovery in non-model organisms (Gaur et al. 2012; Novaes et al. 2008; Pootakham et al. 2013). Nonetheless, discovering and profiling a large number of SNP loci was only possible for crop species with well-established genomic resources until recently. Despite a drastic reduction in the cost associated with next-generation sequencing, it is still prohibitively expensive to employ whole genome sequencing for SNP discovery and genotyping of multiple individuals in a target population, especially for organisms with large genomes.

Reduced representation sequencing has emerged as a method of choice for a wide range of genetic studies, not only because of its cost-effectiveness but also because many research questions can be answered with a small set of markers and do not require whole genome sequencing (Peterson 2005). A recently developed genotyping-by-sequencing (GBS) approach employs restriction endonucleases to ensure that homologous portions of the genome from multiple individuals are targeted for sequencing (Elshire et al. 2011). GBS has been demonstrated to be quick, affordable, and highly robust across a range of species (Gao et al. 2015; Poland et al. 2013; Pootakham et al. 2015b; Russell et al. 2013). The key advantage of GBS is that the technique can be applied to crop species with poorly characterized genome. However, its lack of specificity to the expressed regions of the genome results in a significant portion of sequences originating from non-informative or repetitive regions.

For applications in marker-trait association analyses, SNP markers located in genic regions are generally more informative than those in the intergenic sequences as they are more likely to be in the vicinity of the quantitative loci (QTL) associated with traits of interest. The choice of restriction enzymes influences the number and position of fragments represented in GBS libraries, which in turn affects the number and genomic location of SNPs discovered. The ability to enrich for genic SNPs is often desirable in any dataset and particularly beneficial for trait-association analyses such as QTL mapping, genome-wide association studies (GWAS), and genomic selection. In plants, transposable and repetitive elements are heavily methylated, while the euchromatic regions exhibit lower degrees of cytosine methylation (Zhang et al. 2010). Methylation-sensitive restriction enzymes have been shown to be effective in enriching genomic DNA for gene-containing regions and reducing genomic clones with repeat elements (Fellers 2008).

Depending on the level of complexity reduction applied, GBS datasets can have significant amount of missing data due to low coverage sequencing. Common cutters (four cutters) often produce a large number of small fragments, resulting in a library with low read depth per locus. The use of rare-cutting restriction endonucleases (six cutters) with methylation sensitivity can assist in creating a higher level of complexity reduction by targeting fewer sites, which will lead to higher sampling depth of homologous regions in the genome and reduce the amount of missing data. Here, we evaluated the use of multiple enzyme combinations with and without an additional size selection step to illustrate that GBS protocol can be tailored to achieve varying degrees of complexity reduction. We also demonstrated the effectiveness of methylation-sensitive enzymes in the enrichment of scorable SNPs in genic regions. We used oil palm (*Elaeis guineensis*) in our study as it represents an outcrossing, highly heterozygous, non-model species with a relatively large genome (1.8 Gb) (Singh et al. 2013).

Genomic DNA from two parental cultivars (clone A43/9 and clone A) was used to prepare reduced representation libraries, following the modified GBS protocol using two enzymes and a Y-adapter (Mascher et al. 2013; Pootakham et al. 2015a). DNA samples were digested using various combinations of methylation-sensitive (*Aat*II, *Pst*I and *Msp*I) and methylation-insensitive (*Sph*I and *Mse*I) enzymes (Table 1). To enable multiplex sequencing of the libraries, the forward adapters contained 9-bp unique barcodes in addition to 21 bp of the Ion Forward adapter and a restriction site. The reverse adapter (Y-adapter) contained the ion reverse priming site and was designed such that amplification of the more common fragments generated by the four cutters (*Msp*I-*Msp*I or *Mse*I-*Mse*I) was prevented. We multiplexed 12 samples per run. For size-selected libraries, we selected fragments of ∼270 bp using the E-Gel® SizeSelect™ Agarose Gels (Thermo Fisher, Waltham, MA, USA). Fragment size distribution of the PCR-amplified libraries prior to and after the size selection step is shown in Supplemental Fig. 1. The libraries were quantified using the 2100 Bioanalyzer High Sensitivity DNA kit (Agilent Technologies, Santa Clara, CA, USA) and sequenced on two Ion Proton PI™ Chips according to the manufacturer’s protocol (Thermo Fisher, Waltham, MA, USA). Clean reads were mapped to the oil palm reference genome (Singh et al. 2013) using the Ion Torrent™ Suite Software Alignment Plugin (Torrent Mapping Alignment Program version 5.0.13), and the variants were called using the Ion Torrent Variant Caller (GATK v3.4-46; Thermo Fisher, Waltham, MA, USA). The following (default) parameter setting was applied: minimum sequence match on both sides of the variants – 5; minimum support for a variant to be evaluated – 6; minimum frequency of the variant to be reported – 0.15; and maximum relative strand bias – 0.8. The location of SNPs was analyzed using SNPEff software (Cingolani et al. 2012) with oil palm reference genome sequence and GFF annotation input files (Singh et al. 2013).

**Table 1:** Recognition sites and cytosine methyl-sensitivity of selected restriction endonucleases

Enzyme	Recognition site	Methyl-sensitivity	Cleavage blocked by
*Aat*II	GACGTC	Yes	GA^5m^CGTC
*Pst*I	CTGCAG	Yes	^5m^CTGCAG
*Sph*I	GCATGC	No	Not sensitive to cytosine methylation
*Msp*I	CCGG	Yes	^5m^CCGG
*Mse*I	TTAA	No	Not sensitive to cytosine methylation

Reduced representation libraries were constructed from genomic DNA using six different combinations of methylation-sensitive and methylation-insensitive enzymes (*Aat*II/*Msp*I, *Aat*II/*Mse*I, *Pst*I/*Msp*I, *Pst*I/*Mse*I, *Sph*I/*Msp*I, and *Sph*I/*Mse*I) and sequenced in multiplex on Ion Proton PI™ chips. We obtained a total of 132,965,081 reads covering roughly 15.08 Gb of sequence data, with an average of 5,540,212 reads per sample (Supplemental Table 1). On average, 83% of the total bases sequenced had a quality score of at least 20, and we were able to align approximately 90% of cleaned reads to the publicly available reference genome. SNPs were called using GATK version 3.4-46 with a default parameter setting (McKenna et al. 2010). Only reads that could be mapped to unique locations were used for SNP calling.

The original GBS protocol developed by Elshire et al. (2011) did not include a size selection step and relied solely on the PCR condition that favored the amplification of smaller fragments. Consequently, the size distribution of fragments in GBS sequencing libraries is often less well defined than in libraries derived from other methods with a size selection step (Supplemental Fig. 1). To minimize the amount of missing data in GBS, it is necessary to increase the overall read depth and reduce the level of genome sampling. We investigated the efficiency of various restriction enzymes in reducing genome complexity and whether adding a size selection step to the GBS protocol could achieve a greater level of complexity reduction. After PCR amplification of the pooled libraries, samples were divided into two aliquots and one was size selected on an agarose gel to narrow the fragment pool prior to sequencing. We calculated the number of loci covered at different read depths for size-selected (SS) and non-size-selected (NS) libraries constructed from six enzyme combinations (Fig. 1a). When a methylation-sensitive six-cutter (*Pst*I or *Aat*II) was coupled with a four-cutter that also exhibited methyl-sensitivity (*Msp*I), the numbers of loci obtained from both NS and SS libraries were essentially the same across all read depths examined. The additional size selection step does not seem to be necessary when a pair of methylation-sensitive enzymes (*Pst*I/*Msp*I or *Aat*II/*Msp*I) is used to generate the sequencing libraries.

**Fig. 1:**
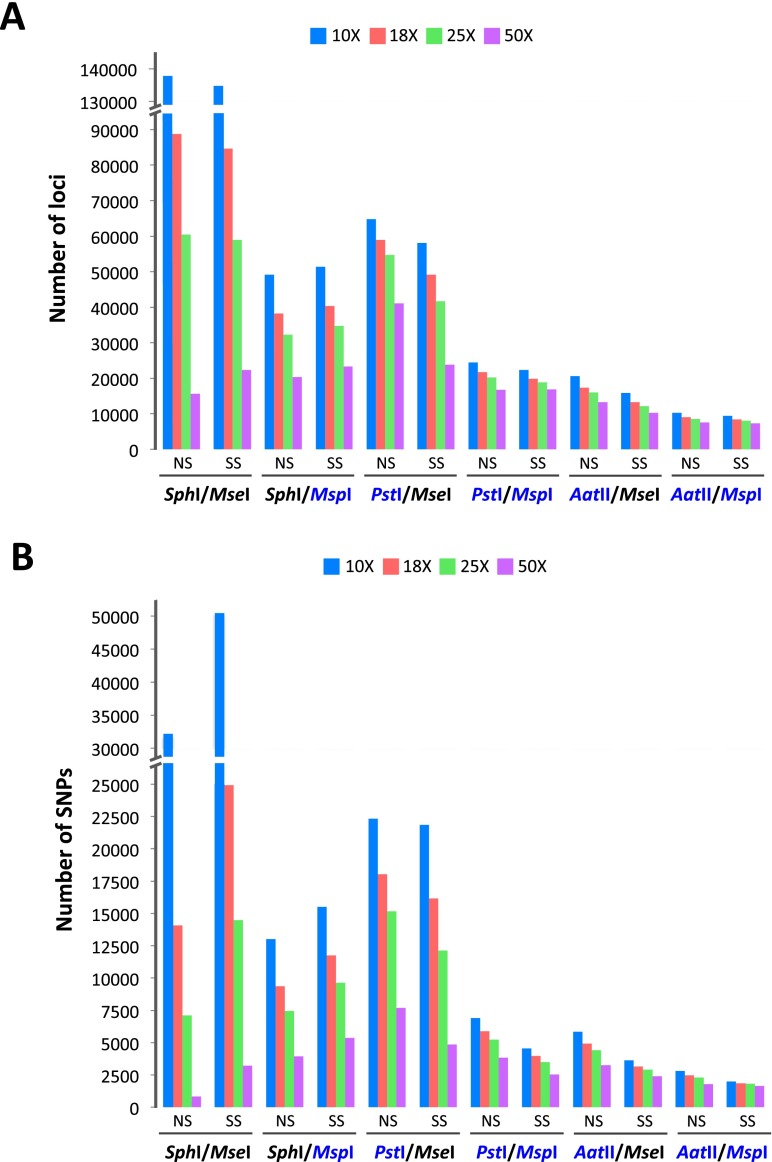
**a** Genome coverage in terms of number of loci covered and **b** number of SNPs identified under various conditions at four read depths (10×, 18×, 25×, and 50×). Methylation-sensitive enzymes are highlighted in *blue*. NS designates non-size-selected libraries while SS designates size-selected libraries (color figure online)

When a methylation-sensitive rare cutter (*Pst*I or *Aat*II) was used in combination with a methylation-insensitive frequent cutter *Mse*I, the additional size selection step significantly reduced the genome coverage by 20–40% (Fig. 1a). Interestingly, the use of a methylation-insensitive six-cutter *Sph*I with either *Msp*I or *Mse*I resulted in higher numbers of loci covered in the SS libraries at greater read depths (50×; Fig. 1a). The ability of *Sph*I and *Mse*I to digest DNA uniformly throughout the genome without any bias toward methylated sequences led to the generation of an enormous number of small fragments, most of which were sequenced at low read depths (10–18×). In this case, the additional size selection step significantly improved the level of genome complexity reduction and as a result, SS libraries yielded higher number of sites covered at >50× read depths. Although the *Sph*I/*Mse*I combination provided great genome coverage at lower depth (10–25×), there was a precipitous decline in the number of loci detected as the read depths increased to 50×, indicating uneven distribution of reads across the genome. Previous estimates of sequencing depth required to accurately call SNPs in whole genome sequencing are variable. Bentley et al. (2008) suggested a minimum of 33× mapped read depth for detection of heterozygous SNPs, while 50× was estimated by Ajay et al. (2011) for all SNPs and small indels (Ajay et al. 2011; Bentley et al. 2008). In order to achieve uniform distribution of reads across the genome and sufficient read depth especially for identifying heterozygous genotypes, we recommend using at least one methylation-sensitive enzyme in GBS library construction. In a situation where methylation-insensitive enzymes are preferred, the additional size selection step may be necessary to improve the overall read depth and the degree of overlap across samples.

Even when the choice of enzymes is geared towards increasing overall GBS read depth, there may be circumstances in which a high level of sample multiplexing is required (e.g., budget constraint), and the average read depth per sample is inevitably diluted. When the GBS data contain a discernable proportion of uncalled genotypes owing to low read coverage, several methods can be employed for the imputation of those missing data (Huang et al. 2014a; Swarts et al. 2014). A number of imputation pipelines have been developed for organisms ranging from an inbred diploid species with a well-characterized genome (such as rice) to a highly heterozygous polyploid species with no reference sequences (such as alfalfa) (Nazzicari et al. 2016).

The number of SNPs identified and the number of mapped loci in each library are generally in congruent (Fig. 1a, b). There is a trade-off between genome coverage and read depth, and the choice of restriction endonucleases depends on the number of SNPs desired and the anticipated level of multiplexing. Although the use of two methylation-sensitive enzymes yields a smaller number of scorable SNPs, it allows a higher degree of multiplexing, hence reducing the cost of sequencing per sample. A combination of methylation-sensitive rare- and common-cutting enzymes, such as *Pst*I/*Msp*I and *Aat*II/*Msp*I, resulted in greater uniformity of read depth across loci and provided much higher quality genotype information. With a range of enzymes evaluated, the level of complexity reduction can be adjusted depending on the appropriate number of SNPs required in each application. Construction of linkage maps for marker-trait association studies may be performed with a few thousands to tens of thousands of SNPs, while the generation of ultra-high density genetic maps for anchoring and ordering physical maps requires dense sets of several hundreds of thousands of markers.

For QTL mapping and association studies, SNP markers located in the expressed portion of the genome are often more informative than those in the intergenic regions. Methylation-sensitive restriction endonucleases have been employed in the construction of reduced representation libraries in order to enrich for hypomethylated gene space and avoid repetitive regions of the genome (Emberton et al. 2005; Nelson et al. 2008). Here, we investigated the effectiveness of methylation-sensitive enzymes in the enrichment of genic SNPs in the oil palm genome. Sequencing data from NS and SS libraries that were constructed from the same pair of enzymes were combined and analyzed together. To obtain high quality SNPs, we filtered an initial set of SNPs on the basis of read depth (minimum 25 reads/SNP/individual). Each of the filtered SNP was subsequently categorized into three groups: “gene” if it was located within the annotated genes, “gene ± 5 kb” if it was located within 5 kb either upstream or downstream from annotated genes, and “intergenic” if it did not fit either of the above criteria. Genomic distribution of SNPs identified from six enzyme combinations is shown in Fig. 2. When a pair of methylation-insensitive enzymes (*Sph*I/*Mse*I) was employed in GBS library construction, only 16% of SNP loci were located in genes and 18% in the vicinity (± 5 kb) of the genic regions, while the remaining SNPs (66%) resided in the intergenic regions. A combination of methylation-sensitive and methylation-insensitive endonucleases (*Sph*I/*Msp*I and *Aat*II/*Mse*I) yielded approximately 20% genic SNPs. Notably, almost two thirds of total SNPs discovered in the reduced representation libraries generated with two methylation-sensitive enzymes (*Aat*II/*Msp*I and *Pst*I/*Msp*I) were located either inside (32–36%) or in the vicinity (28–31%) of the genic regions. Interestingly, the use of *Pst*I revealed a high percentage of SNPs (∼36%) located in gene space regardless of the methyl-sensitivity of its common-cutting enzyme partner, while an apparent 10% difference in the number of genic SNPs was observed between the *Aat*II/*Msp*I and *Aat*II/*Mse*I libraries.

**Fig. 2:**
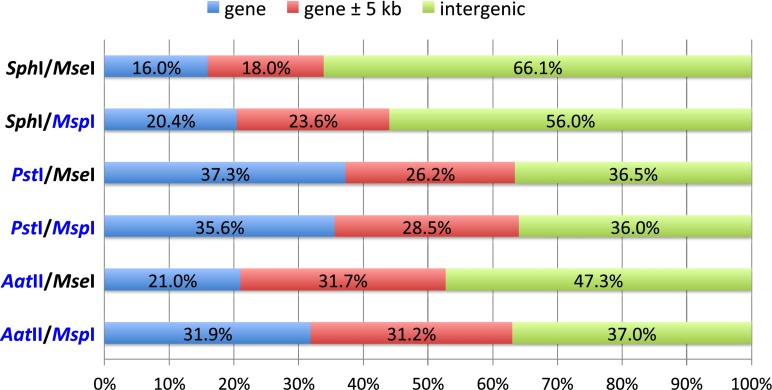
Distribution of SNPs in the genome. Methylation-sensitive enzymes are highlighted in *blue* (color figure online)

Of the six enzyme combinations evaluated, *Pst*I/*Msp*I and *Pst*I/*Mse*I appeared to be most effective in capturing fragments that are rich in gene-containing sequences in oil palm. Both *Pst*I and *Msp*I are sensitive to CNG methylation, whereas *Aat*II is sensitive to CG methylation (Gruenbaum et al. 1981). A significant degree of enrichment in genic SNPs and SNPs located within 5 kb of coding regions observed in both *Pst*I/*Msp*I and *Pst*I/*Mse*I libraries suggested that, in oil palm, substantial portions of the CNG trinucleotides in intergenic regions are predominant targets of methylation, while the proportion of methylated CG dinucleotide sites in intergenic sequences may be smaller. Since both *Pst*I/*Msp*I and *Pst*I/*Mse*I libraries yielded a remarkable level of enrichment in gene space, we recommended genotyping oil palm mapping population using either of these pairs for reduced representation libraries construction. The degree of genic SNP enrichment may vary among plant species and how well each methylation-sensitive enzyme contributes to such enrichment may have to be determined empirically. Frequency distributions of SNPs along oil palm chromosomes revealed that a pair of methylation-insensitive enzymes (*Sph*I/*Mse*I) yielded a relatively uniform SNP distribution across the physical map (Supplemental Fig. 2). On the other hand, SNPs discovered using combinations of two methylation-sensitive enzymes (*Pst*I/*Msp*I and *Aat*II/*Msp*I) tended to cluster in non-repetitive regions of the genome (Supplemental Fig. 2) (Singh et al. 2013).

Taking genome coverage data (Fig. 1) into consideration, *Pst*I/*Mse*I libraries showed a higher coverage of mapped reads, whereas the *Pst*I/*Msp*I libraries offered a greater level of complexity reduction. While *Pst*I and *Msp*I appear to be enzymes of choice in several species (Huang et al. 2014b; Poland et al. 2013; Sonah et al. 2013), the use of *Pst*I and *Mse*I may be more suitable for projects that require higher genome coverage. In certain situations where a uniform distribution of markers including those in the methylated regions of the genome is desired, the use of methylation-insensitive enzymes may be preferable. Under those circumstances, the additional size selection step should be applied to ensure sufficient depth coverage for SNP calling. The choice of enzymes can be adjusted to increase the coverage of the target genome or the multiplexing level to achieve an optimal condition for the species under investigation. For outcrossing species, obtaining sufficient read depth at each locus is crucial for accurate calling of heterozygous genotypes. We hope that our evaluation of various enzyme combinations will provide useful information to help scientists and molecular breeders optimize GBS protocols for their species of interest.

## Electronic supplementary material

Supplemental Figure 1 (PDF 526 kb)

Supplemental Figure 2 (PDF 646 kb)

Supplemental Table 1 (XLSX 183 kb)
