# Supplementary material for: Effects of methylation-sensitive enzymes on the enrichment of genic SNPs and the degree of genome complexity reduction in a two-enzyme genotyping-by-sequencing (GBS) approach: a case study in oil palm (Elaeis guineensis)
Source: Mol Breed. 2016 Nov 10;36(11):154. doi: 10.1007/s11032-016-0572-x (PMC5104780; doi:10.1007/s11032-016-0572-x)
Supplement: Supplementary file 1 — (PDF 526 kb) [file 11032_2016_572_MOESM1_ESM.pdf]

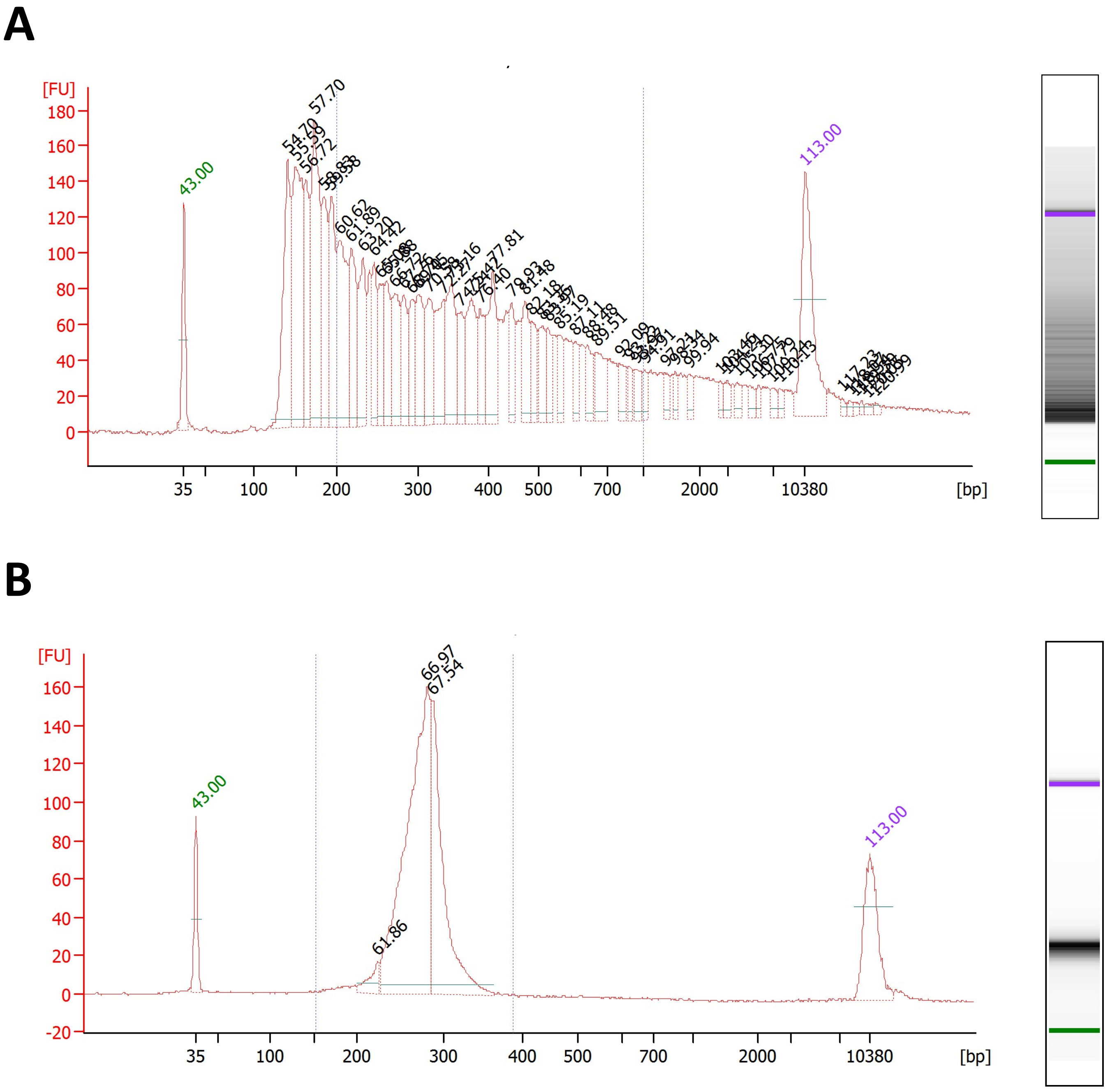

Supplemental Fig. 1 Bioanalyzer traces showing the size distribution of the PCR-amplified library (A) prior to and (B) after a size selection step on the 2% E-gel. The size of fragments selected was approximately 270 bp.
