# Supplementary material for: Effects of methylation-sensitive enzymes on the enrichment of genic SNPs and the degree of genome complexity reduction in a two-enzyme genotyping-by-sequencing (GBS) approach: a case study in oil palm (Elaeis guineensis)
Source: Mol Breed. 2016 Nov 10;36(11):154. doi: 10.1007/s11032-016-0572-x (PMC5104780; doi:10.1007/s11032-016-0572-x)
Supplement: Supplementary file 2 — (PDF 646 kb) [file 11032_2016_572_MOESM2_ESM.pdf]

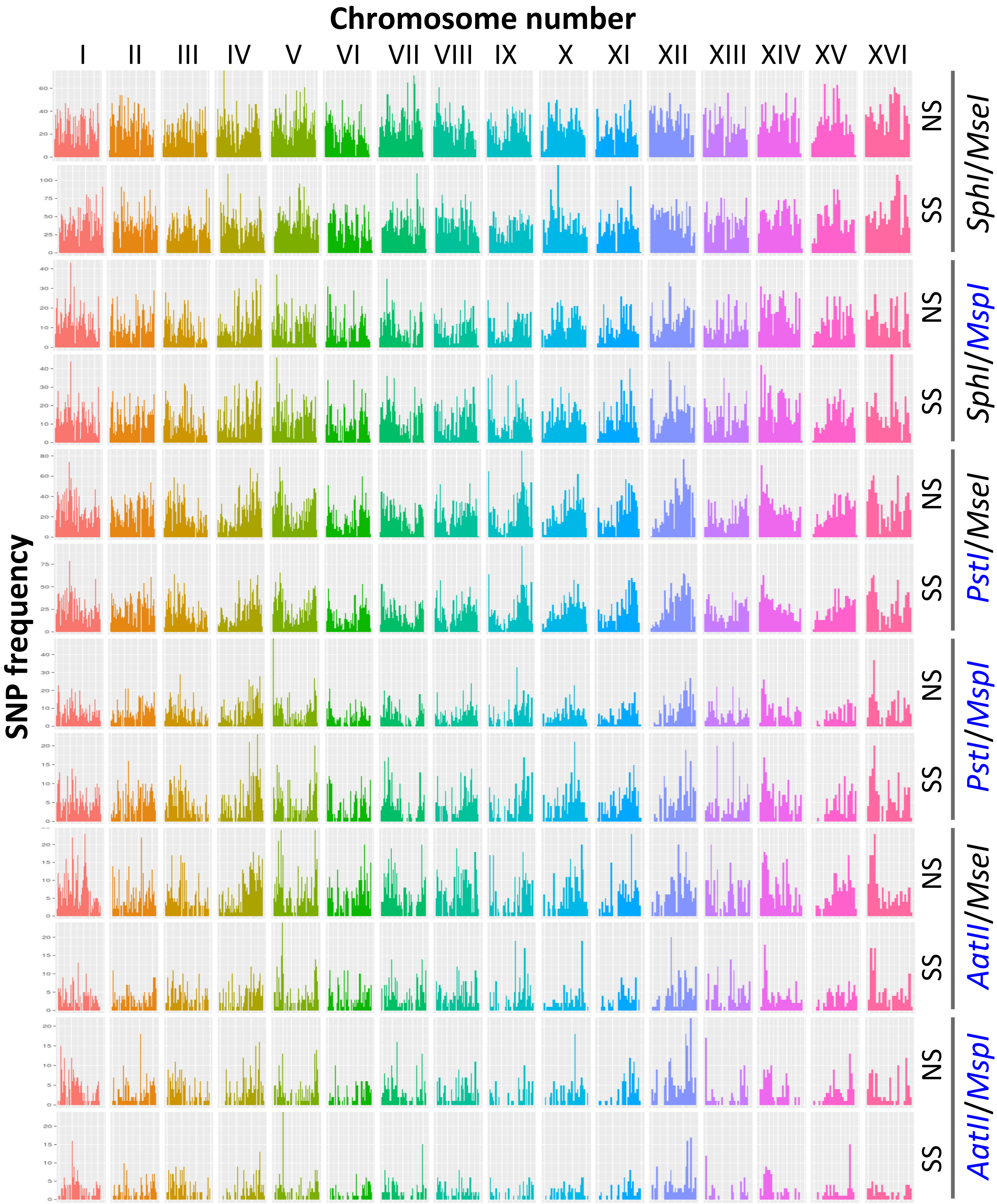

Supplemental Figure 2. Frequency distribution of SNPs along oil palm chromosomes. SNPs were identified using different combinations of methylation-sensitive (highlighted in blue) and methylation-insensitive enzymes at the sequence depth of at least 10 reads/SNP/ sample. NS and SS indicate whether size selection has been applied (NS – no size selection; SS – size selection).
